# Association among epicardial fat, heart rate recovery and circadian blood pressure variability in patients with hypertension

**DOI:** 10.1186/s40885-015-0034-5

**Published:** 2015-11-22

**Authors:** Da-Jung Kim, Kyoung-Im Cho, Eun-A Cho, Jin-Wook Lee, Hyun-Joon Park, Sun-Min Kim, Hyun-Su Kim, Jung Ho Heo

**Affiliations:** Division of Cardiology, Department of Internal Medicine, Kosin University College of Medicine, 34, Amnam-dong, Seo-gu, Busan, 602-702 Korea

**Keywords:** Epicardial fat, Cardiac autonomic function, Heart rate recovery, Hypertension

## Abstract

**Background:**

Epicardial fat tissue is known to have an unique endocrine function which affect the cardiac autonomic system. Heart rate recovery (HRR) is a simple non-invasive measurement that assesses autonomic nervous system dysfunction. We aimed to investigate the association among epicardial fat thickness (EFT), HRR and circadian blood pressure (BP) variation in patients with hypertension.

**Methods:**

A total of 358 consecutive patients who underwent both 24-hour ambulatory BP monitoring (ABPM) and a treadmill test were enrolled. Echocardiographic EFT and HRR, defined as peak heart rate minus heart rate after a 1-min recovery time, were measured. Patients were classified according to the ABPM; 147 patients with hypertension with a dipping pattern at night (dippers), 140 patients with hypertension with a non-dipping pattern at night (non-dippers) and 71 normotensive controls.

**Results:**

EFT was significantly higher in hypertensive patients, especially in the non-dipper group, compared to the controls (non-dippers, 7.5 ± 2.9 mm; dippers, 6.6 ± 1.6 mm; controls, 5.5 ± 2.1 mm; *p* < 0.001). HRR was significantly lower in both hypertensive groups as compared to the control group and was the lowest in the non-dipper group (non-dipper, 26.6 ± 18.6; dipper, 29.5 ± 21.5; control, 71.4 ± 19.8; *p* < 0.001). EFT was significantly correlated with age, body mass index, 24-hour mean systolic BP and 24 h mean BP variability, whereas exercise duration, metabolic equivalents (METs) and HRR were inversely correlated with EFT. Furthermore, EFT > 6.7 mm was associated with a blunted HRR with 76 % sensitivity and 61 % specificity (ROC area under curve: 0.71, 95 % confidence interval, CI = 0.65–0.76, *p* < 0.001). In a multivariate analysis, EFT (odds ratio, OR = 3.53, 95 % CI = 1.20–10.37, *p* = 0.022) and 24-hour mean BP variability (OR = 1.09, 95 % CI = 1.03–1.16, *p* = 0.005) were independent predictors of a blunted HRR defined as HRR ≤ 12 beats (*n* = 63) in patients with hypertension.

**Conclusion:**

EFT and HRR were significantly correlated with circadian BP variability in patients with hypertension. EFT and circadian BP variability were independent predictors of blunted HRR, which suggests a link between epicardial fat and autonomic dysregulation in hypertension.

## Background

Blood pressure (BP) is subject to diurnal variation, and studies using ambulatory BP monitoring (ABPM) have demonstrated that a blunted reduction in nocturnal BP (i.e., a non-dipping pattern) is associated with severe end-organ damage and an increased risk of cardiovascular events, especially in hypertensive patients [1, 2]. Although pathologic mechanisms are still unclear, non-dippers are suggested to show the impairment in the autonomic system functions that include abnormal sympathetic and parasympathetic activities [3, 4]. Heart rate recovery (HRR) is a simple non-invasive measurement analyzing autonomic nervous system dysfunction, which indicates impaired parasympathetic reactivation [5–7]. HRR after exercise is emerging as a new and important prognostic index, [8, 9] and an earlier study showed that a blunted HRR defined as a decrease in heart rate (HR) from peak exercise to 1 min into recovery of ≤ 12 beats/min is a powerful predictor of overall mortality [10]. Recently, it has been shown that blunted HRR is common in patients with hypertension, and this phenomenon is associated with cardiovascular risk [5].

Epicardial fat thickness (EFT) is a newly identified cardiovascular risk factor. A high amount of epicardial fat is dangerous because this fat tissue is known to have unique endocrine and paracrine functions which affect the cardiac autonomic system [11–13]. Because elevated BP is associated with ectopic fat accumulation in the intrathoracic and epicardial areas, an association between epicardial adipose tissue and hypertension [14, 15], as well as with diurnal BP patterns, [16, 17] has been suggested in some recent studies. However, the association between EFT and autonomic function assessed by HRR in patients with hypertension has not been well studied. So, we aimed to investigate the association among EFT, HRR and circadian BP variation in patients with hypertension.

## Methods

### Study population

This cross-sectional, observational single-center cohort study included 358 consecutive patients who simultaneously underwent 24-hour ABPM, an exercise treadmill test and echocardiography between January 2010 and March 2015. Inclusion criteria were: 18–80 years of age, normal renal function and for women to be on a regular menstrual cycle. Exclusion criteria were: any systemic disease such as significant liver disease, neurologic disorders or malignant disease; secondary hypertension; valvular heart disease; a positive treadmill test; a history of heart failure; a history of acute coronary syndrome; myocar dial infarction or any revascularization procedure. Demographic characteristics recorded at the first visit included age, gender, height, weight, current medications, smoking history and other comorbidities. Blood was drawn for measurement of total serum cholesterol, high-density lipoprotein (HDL), low-density lipoprotein (LDL) cholesterol, triglycerides, blood glucose, creatinine, uric acid, and high sensitivity C-reactive protein (hs-CRP). Body mass index (BMI) was calculated as the ratio of weight in kilograms to height in square meters. This study was approved by the Kosin University International Review Board. All patients were required to provide written informed consent to participate.

### Blood pressure measurement and ambulatory blood pressure monitoring

Office BP measurements were measured twice at 5-min intervals using a mercury sphygmomanometer. Noninvasive 24-hour ABPM was performed on each patient’s non-dominant arm using an automatic oscillometric device (TONOPORT V, PAR Medizintechnik, Berlin, Germany) on a normal working day. Patients were generally asked to refrain from fast exercise or stop taking the antihypertensive medications before 48 h. All subjects were instructed to rest or sleep between 10:00 PM and 7:00 AM (nighttime) and to continue their usual activities between 7:00 AM and 10:00 PM (daytime). The accuracy of the device was checked against the standard auscultatory method to assure the difference in BP measurements between methods did not exceed 5 mmHg. The device was set to obtain BP readings at 20-min intervals during the daytime and at 40-min intervals during the nighttime. Only 24-hour recordings that included at least 80 % successful recordings were accepted as valid. Each ABPM dataset was first automatically scanned to remove artifactual readings according to preselected editing criteria. The following ABPM parameters were evaluated: 24-hour mean systolic and diastolic BP levels, daytime mean systolic and diastolic BP levels, nighttime mean systolic and diastolic BP levels and BP variability assessed by standard deviation (SD). Additionally, the magnitude of the nocturnal decline in BP (Δ nocturnal decline) was calculated as follows: daytime average BP minus nighttime average; the percentage change in BP from day to night (% day – night BP) was calculated as: (daytime BP – nighttime BP) × 100/daytime BP.

### Diagnosis of hypertension

Following the recommendations of the European Society of Hypertension, [18] a normotensive state was defined as a mean daytime ambulatory systolic and diastolic BP < 135/85 mmHg by ABPM, associated with an office BP < 140/90 mmHg. True hypertension was assigned if the average daytime BP was higher than 135/85 mmHg and the average nighttime BP was above 120/75 mmHg. In addition, the hypertensive subjects who had reduction in BP < 10 % change from daytime to nighttime period were defined as “non-dippers”, and the hypertensive subjects who had a reduction in BP ≥ 10 % change from daytime to nighttime were considered “dippers”. Patients were classified according to the ABPM; 147 patients had hypertension and the dipping pattern (dippers), 140 patients had hypertension and a non-dipping pattern (non-dippers) and 71 were normotensive controls.

### Echocardiographic measurement

**Fig. 1:**
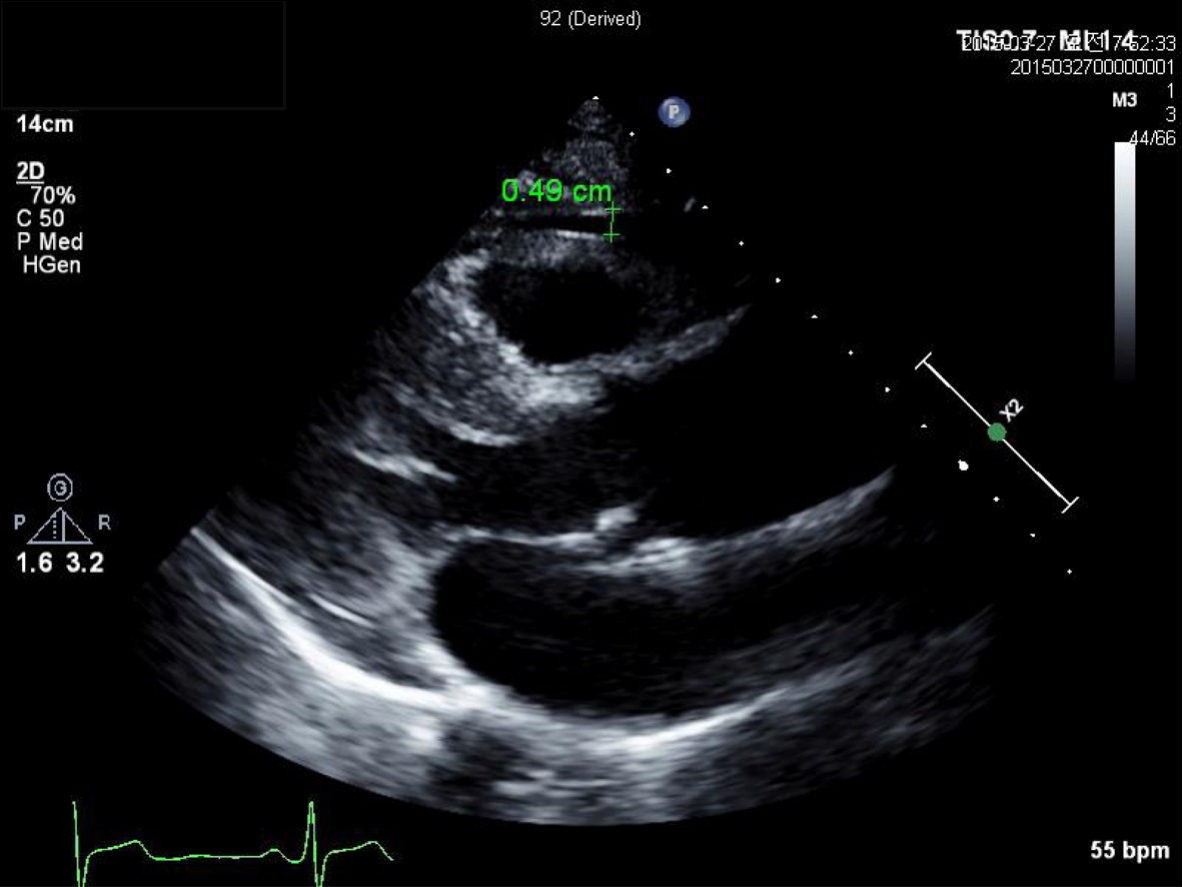
Echocardiographic assessments of EFT, defined as the echo-free space between the outer wall of the myocardium and the visceral layer of the pericardium, were measured perpendicularly from the free wall of the right ventricle at end-systole in the parasternal long axis view

Standard 2-dimensional echocardiography were performed on all subjects while lying in the left lateral decubitus position using a 3.5-MHz transducer (Philips iE33, Philips Medical Systems, Bothell, WA, USA) and the echocardiography examiners were blinded to patient information. Measurements of the thickness of the interventricular septum and posterior wall, the diameter of the left ventricle (LV) cavity, and the LV mass index (LVMI) were performed according to criteria outlined by the American Society of Echocardiography [19]. Echocardiographic assessments of EFT, defined as the echo-free space between the outer wall of the myocardium and the visceral layer of the pericardium, were measured perpendicularly from the free wall of the right ventricle at the end-systole in three cardiac cycles according to the method we previously described (Fig. 1) [20]. Because one of the critical issues in EFT measurement is the inconsistency in the measurement location, and mean EFT was averaged from the images of the parasternal long axis, parasternal short axis and apical 4 chamber view. Independent offline measurement of EFT was performed by two cardiologists (DJ Kim and KI Cho) who were unaware of the clinical data in the first 50 continuous patients, which was repeated at least twice. A reliability analysis using intra-class correlation coefficient was performed to obtain the intra-observer and inter-observer variability. The intra- and inter-observer variability of the EFT was 3.3 and 5.8 %, respectively.

### Exercise treadmill testing

On the same day as the echocardiographic examination, patients underwent symptom-limited exercise stress testing (GE CASE T2100; GE Medical Systems, Milwaukee, WI, USA) according to the protocol by Bruce et al. [21]. BP was measured with an automated BP monitor (Suntech Tango; Suntech Medical, Morrisville, NC, USA) throughout the treadmill test using the same arm as resting BP was measured on. Twelve-lead electrocardiography was monitored continuously and was printed at a paper speed of 25 mm/s; measurements of HR and BP were recorded at the end of each 3-min stage, at peak exercise and at 1-min intervals throughout recovery. The participants continued to exercise until volitional fatigue or if their HR exceeded 95 % of estimated maximal HR (220 bpm - age). Total exercise time was also recorded. Functional capacity was estimated in metabolic equivalents (METs) on the basis of the spceed and grade of the treadmill [22]. During the recovery phase, the subjects continued to walk for 60 s at a speed of 1.5 mph, and then they sat down for 3 min with continued monitoring of BP, HR and heart rhythm. The value for the HRR was defined as the decrease in the HR from peak exercise to one minute after the cessation of exercise. An abnormal value for the HRR was defined as ≤ 12 beats/min in accordance with previous studies [10].

### Statistical analysis

Statistical analyses were performed with the commercially available computer program SPSS 18.0 for Windows (SPSS Inc., Chicago, IL, USA). Data are presented as mean ± standard deviation for continuous variables and their percentages (%) if the data are categorical. The Mann – Whitney U test was used for continuous variables and the chi-square test was used for categorical data. The normality of data was tested using the Kolmogorov–Smirnov test. Parameter differences among the three groups were evaluated using a one-way ANOVA for normally distributed variables or the Kruskal–Wallis test for non-normally distributed variables. Relationships between variables were examined with Pearson correlation coefficients. The cutoff value of EFT for predicting blunted HRR with corresponding sensitivity and specificity was estimated by receiving operator characteristic (ROC) curve analysis. Multivariate logistic regression models for blunted HRR were built to determine which variables were independently associated with this status. A two-tailed *p* < 0.05 was considered to be statistically significant.

## Results

### Comparison of clinical and ambulatory blood pressure monitoring parameters

A total of 287 hypertensive patients (male/female: 158/129 and age: 51.3 ± 14.4) and 71 normotensive patients (controls, male/female: 34/37 and age: 52.0 ± 12.1) were analyzed, and their clinical features and ambulatory blood pressure parameters according to diurnal variation are given in Tables 1 and 2. Although hypertensive patients were more obese and had a higher heart rate, there were no significant differences in the blood chemistry except hemoglobin level and platelet count among the groups. The non-dipper group was more female, and these patients were on more beta blockers and diuretics (all *p* < 0.05). Circadian BP profile and BP variability assessed by 24-hour mean BP SD were greater in hypertensive patients, and especially in non-dippers (all *p* < 0.05).

**Table 1:** Baseline clinical and laboratory characteristics according to the diurnal variation

	Control group (*n* = 71)	Dipper group (*n* = 147)	Non-dipper group (*n* =140)	*p*-value (ANOVA)
Age, years	52.0 ± 12.1	50.4 ± 13.7	52.2 ± 15.0	0.492
Male gender, n (%)	34 (47.9 %)	93 (63.3 %)	65 (46.4 %)**	0.010
Body mass index, kg/m^2^	23.2 ± 2.8	24.7 ± 3.6*	25.2 ± 4.0*	0.001
Office systolic BP, mmHg	124 ± 12.1	131 ± 17.2*	132 ± 17.9*	0.008
Office diastolic BP, mmHg	72.5 ± 10.7	79.3 ± 13.3*	78.5 ± 13.3*	0.002
Heart rate, bpm	64.5 ± 13.3	65.8 ± 9.6	68.2 ± 12.5*	0.033
Current smoking, n (%)	7 (9.6 %)	22 (15.0 %)	19 (13.6 %)	0.422
Diabetes, n (%)	5 (7.0 %)	11 (7.5 %)	12 (8.6 %)	0.908
Dyslipidemia, n (%)	23 (32.4 %)	50 (34.1 %)	55 (39.3 %)	0.077
Previous BP medication				
RAS blockade, n (%)	-	26 (17.7 %)	31 (22.1 %)	0.211
Beta blocker, n (%)	-	17 (11.6 %)	33 (23.6 %)	0.005
Calcium channel blocker, n (%)	-	26 (17.7 %)	36 (25.7 %)	0.065
Diuretics, n (%)	-	7 (4.7 %)	16 (11.4 %)	0.030
Uric acid, mg/L	5.45 ± 1.48	5.65 ± 1.44	5.60 ± 1.35	0.741
eGFR MDRD	100.0 ± 24.2	96.0 ± 23.1	95.4 ± 27.4	0.467
Creatinine, mg/dL	0.78 ± 0.20	0.89 ± 0.51	0.90 ± 0.61	0.331
Fasting glucose, mg/dL	101 ± 14.7	100 ± 25.2	103 ± 23.4	0.645
Total cholesterol, mg/dL	178 ± 44.3	190 ± 43.5	181 ± 38.2	0.124
LDL cholesterol, mg/dL	102 ± 39.3	108 ± 35.6	102 ± 34.7	0.317
HDL cholesterol, mg/dL	46.4 ± 13.7	50.3 ± 13.7	49.7 ± 12.2	0.194
Triglycerides, mg/dL	127 ± 63.0	163 ± 173	149 ± 92.4	0.230
Hs-CRP, mg/dL	0.11 ± 0.15	0.23 ± 1.04	0.39 ± 1.15	0.259
White blood cells, 10^3^/μL	7.05 ± 2.35	6.92 ± 2.06	7.49 ± 2.57	0.117
Hemoglobin, g/dL	13.5 ± 1.5	13.8 ± 1.7	14.2 ± 1.5*	0.008
Hematocrit, %	40.0 ± 4.7	40.6 ± 4.9	41.8 ± 4.6*	0.021
Platelets, 10^3^/μL	217 ± 52.9	226 ± 53.4	237 ± 61.1*	0.028

All values are presented as the mean ± SD. *BP* blood pressure, *RAS* renin angiotensin system, *LDL* low density lipoprotein, *HDL* high density lipoprotein, *Hs-CRP*high sensitivity C-reactive protein; *p < 0.05 vs. normotensive control group, **p < 0.05 vs. dipper group

**Table 2:** Comparison of parameters of 24-hour ambulatory BP monitoring according to the diurnal variation

	Control group (*n* = 71)	Dipper group (*n* = 147)	Non-dipper group (*n* = 140)	*p*-value (ANOVA)
24-hour HR, bpm	70.0 ± 8.1	75.5 ± 10.8*	74.6 ± 11.9*	0.002
24-hour HR SD, bpm	15.4 ± 6.6	16.0 ± 6.0	14.2 ± 6.9	0.064
Daytime HR, bpm	73.4 ± 8.6	79.7 ± 11.6*	76.9 ± 11.9	<0.001
Nighttime HR, bpm	60.9 ± 7.7	65.2 ± 7.9*	65.9 ± 13.4*	<0.001
24-hour mean SBP, mmHg	119.2 ± 7.7	141.1 ± 11.3*	142.2 ± 14.6*	<0.001
24-hour mean DBP, mmHg	74.8 ± 4.8	91.3 ± 9.6*	89.0 ± 9.7*	<0.001
24-hour mean SBP SD, mmHg	13.1 ± 3.5	15.3 ± 4.6*	16.0 ± 3.7*	<0.001
24-hour mean DBP SD, mmHg	10.7 ± 3.2	13.9 ± 3.8*	13.0 ± 4.8*	<0.001
24-hour mean BP, mmHg	89.3 ± 5.2	106.1 ± 10.7*	108.2 ± 9.6*	<0.001
24-hour mean BP variation, mmHg	11.2 ± 3.3	13.4 ± 4.9*	14.0 ± 3.5*	<0.001
Daytime SBP, mmHg	121.1 ± 7.8	146.2 ± 11.7*	143.1 ± 14.4*	<0.001
Daytime DBP, mmHg	76.9 ± 4.8	95.2 ± 9.8*	90.1 ± 9.9*,**	<0.001
Daytime SBP SD, mmHg	12.6 ± 4.1	13.9 ± 4.2	15.2 ± 4.9*,**	<0.001
Daytime DBP SD, mmHg	10.6 ± 3.9	12.5 ± 4.7*	12.8 ± 5.1*	<0.001
Daytime mean BP, mmHg	91.1 ± 5.4	112 ± 9.8*	107 ± 10.8*,**	<0.001
Daytime mean BP SD, mmHg	10.7 ± 4.0	12.2 ± 4.3	12.9 ± 4.8*,**	0.001
Nighttime SBP, mmHg	111 ± 15.5	129 ± 12.1*	139 ± 16.7*,**	<0.001
Nighttime DBP, mmHg	70.1 ± 6.2	80.2 ± 12.0*	85.7 ± 10.7*,**	<0.001
Nighttime SBP SD, mmHg	10.3 ± 3.0	12.2 ± 3.9*	12.8 ± 4.2*	<0.001
Nighttime DBP SD, mmHg	8.5 ± 2.9	10.4 ± 3.7*	10.5 ± 4.5*	<0.001
Nighttime mean BP, mmHg	83.9 ± 6.9	95.8 ± 12.6*	103 ± 11.8*,**	<0.001
Nighttime mean BP SD, mmHg	8.8 ± 2.8	10.6 ± 3.6*	10.7 ± 4.2*,**	<0.001
Day-night difference, mmHg	8.1 ± 7.0	15.1 ± 8.3	3.4 ± 6.1*,**	<0.001

All values are presented as the mean ± SD. *BP* blood pressure, *HR* heart rate, *SBP* systolic blood pressure, *DBP* diastolic blood pressure, *SD* standard deviation, *p < 0.05 vs. normotensive control group, **p < 0.05 vs. dipper group

### Comparison of exercise stress testing and echocardiographic parameters

**Fig. 2:**
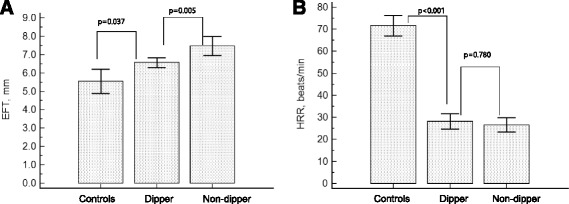
Comparison of epicardial fat thickness (EFT) and heart rate recovery (HRR) according to the hypertension. EFT was significantly higher in hypertensive patients, especially with non-dipper group as compared to controls (**a**). HRR was significantly lower in both hypertensive groups as compared to the control group (**b**)

Although there was no significant difference in systolic function, hypertensive patients showed significantly greater wall thickness, greater LVMI and a larger left atrial diameter, all of which were more prominent among the non-dippers (Table 3). EFT was significantly higher in hypertensive patients, especially in the non-dipper group compared to the control group (non-dippers, 7.5 ± 2.9 mm; dippers, 6.6 ± 1.6 mm; controls, 5.5 ± 2.1 mm; *p* < 0.001; Fig. 2a). A comparison of the results of symptom-limited exercise stress testing is shown in Table 4. Exercise time and METs were significantly lower in the hypertensive patients (both *p* < 0.05). HRR was significantly lower in both hypertensive groups as compared to the control group and was the lowest in the non-dipper group (non-dippers, 26.6 ± 18.6; dippers, 29.5 ± 21.5; controls, 71.4 ± 19.8; *p* < 0.001; Fig. 2b). The incidence of blunted HRR defined as ≤ 12 beats/min was about 30 % in hypertensive patients (*n* = 63); there was no significant difference between dipper and non-dipper groups.

**Table 3:** Comparison of echocardiographic parameters according to the diurnal variation

	Control group (*n* = 71)	Dipper group (*n* = 147)	Non-dipper group (*n* = 140)	*p*-value (ANOVA)
EFT, mm	5.54 ± 2.06	6.57 ± 1.61*	7.47 ± 2.93*,**	<0.001
LVEDD, mm	46.0 ± 5.0	46.0 ± 5.2	45.8 ± 5.4	0.946
LVESD, mm	29.1 ± 4.8	29.0 ± 4.7	29.0 ± 4.7	0.979
IVSTd, mm	11.2 ± 2.2	12.4 ± 2.5*	12.5 ± 2.8*	0.004
PWTd, mm	9.7 ± 2.1	10.5 ± 2.0*	10.7 ± 1.9*	<0.001
LVMI, g/m^2^	103 ± 28.7	112 ± 26.4	114 ± 34.9*	0.044
RWT	0.43 ± 0.11	0.46 ± 0.11	0.47 ± 0.10*	0.046
EF, %	66.7 ± 6.5	66.9 ± 7.1	66.4 ± 7.7	0.839
LA volume, mL	16.8 ± 7.8	16.7 ± 6.4	19.1 ± 7.7*,**	0.022
E velocity, cm/sec	0.67 ± 0.16	0.64 ± 0.14	0.67 ± 0.17	0.389
A velocity, cm/sec	0.67 ± 0.17	0.65 ± 0.17	0.72 ± 0.22**	0.023
EEa	9.4 ± 2.4	8.9 ± 2.6*	11.2 ± 4.8*,**	<0.001

All values are presented as the mean ± SD. EFT, epicardial fat thickness; *LVEDD* left ventricular end-diastolic diameter, *LVESD* left ventricular end-systolic diameter; *IVSTd* diastolic interventricular septal wall thickness; *PWTd*diastolic posterior wall thickness, *LVMI* left ventricular mass index, *RWT* relative wall thickness, *EF* ejection fraction, *LA* left atrial diameter, *E* peak early diastolic mitral filling velocity, *Ea*, mitral annular velocity, *A* peak late diastolic mitral filling velocity, *p < 0.05 vs. normotensive control group, **p < 0.05 vs. dipper group

**Table 4:** Comparison of symptom-limited exercise stress testing according to the diurnal variation

	Control group (*n* = 71)	Dipper group (*n* = 147)	Non-dipper group (*n* = 140)	*p*-value (ANOVA)
Exercise time, min	8.79 ± 2.02	7.84 ± 2.32*	7.69 ± 2.39*	<0.001
Metabolic equivalents	10.7 ± 2.3	9.7 ± 2.6*	9.4 ± 2.6*	<0.001
Rest heart rate, bpm	64.5 ± 13.3	65.8 ± 9.6	68.2 ± 12.5	0.061
Max heart rate, bpm	156 ± 26.4	162 ± 19.6	155 ± 24.2	0.064
HRR, bpm	71.7 ± 19.8	28.3 ± 21.1*	26.6 ± 19.4*	<0.001
Blunted HRR	0	43 (29.3 %)*	42 (30 %)*	<0.001
Rest systolic BP, mmHg	117 ± 11.5	135 ± 14.6*	134 ± 16.7*	<0.001
Rest diastolic BP, mmHg	70.7 ± 10.0	78.4 ± 14.3*	78.1 ± 13.1*	<0.001
Max systolic BP, mmHg	166 ± 23.1	185 ± 20.9*	181 ± 24.8*	<0.001
Max diastolic BP, mmHg	79.7 ± 12.9	86.5 ± 13.8*	85.5 ± 16.4*	0.005

All values are presented as the mean ± SD. *HR* heart rate reserve, *BP* blood pressure, *p < 0.05 vs. normotensive control group, *p* < 0.05 vs. dipper group

### Correlations between HRR or EFT and clinical parameters

**Fig. 3:**
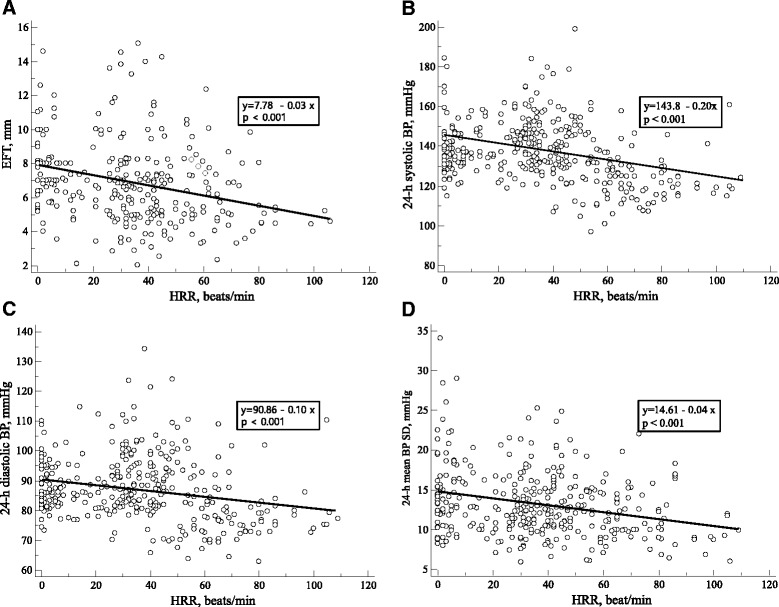
Correlations between epicardial fat thickness (EFT) or heart rate recovery (HRR) and clinical parameters. HRR was significantly negatively correlated with EFT (**a**), 24-hour mean systolic BP (**b**), 24-hour mean diastolic BP (**c**), and 24-hour mean BP SD (**d**)

**Fig. 4:**
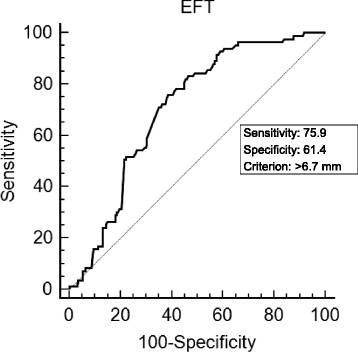
Receiving operator characteristic curve analysis of cutoff value of epicardial fat thickness (EFT) for predicting blunted heart rate reserve (HRR). EFT > 6.7 mm was associated with the blunted HRR with 76 % sensitivity and 61 % specificity

HRR was significantly negatively correlated with EFT (r = −0.309, *p* < 0.001, Fig. 3a), 24-hour mean systolic BP (r = −0.343, *p* < 0.001, Fig. 3b), 24-hour mean diastolic BP (r = −0.255, *p* < 0.001, Fig. 3c) and 24-hour mean BP SD (r = −0.251, *p* < 0.001, Fig. 3d). Moreover, EFT was significantly positively correlated with age, BMI, 24-hour mean systolic BP and 24 h mean BP SD; exercise duration and METs were inversely correlated with EFT (all *p* < 0.05, Table 5). Furthermore, EFT > 6.7 mm was associated with blunted HRR with 76 % sensitivity and 61 % specificity (ROC area under curve: 0.71, 95 % confidence interval = 0.65–0.76, *p* < 0.001) (Fig. 4). In multivariate analysis, EFT (odds ratio, OR = 3.53, 95 % confidence interval, CI =1.20–10.37, *p* = 0.022) and 24-hour mean BP SD (OR = 1.09, 95 % CI = 1.03–1.16, *p* = 0.005) were independent predictors of blunted HRR in patients with hypertension (Table 6). When we performed sensitivity analysis for the multivariate regression analysis according to the use or nonuse of medications, especially beta blockers, the results were not different.

**Table 5:** Correlations between heart rate recovery (HRR) or epicardial fat thickness (EFT) and clinical parameters in the study groups

	HRR		Blunted HRR		EFT	
	r	p	r	p	r	p
Age, years	−0.033	0.541	0.041	0.443	0.123	0.035
Female gender	0.083	0.120	−0.045	0.397	0.133	0.021
Body mass index	−0.078	0.145	0.003	0.960	0.110	0.040
Left ventricular mass index	−0.023	0.686	−0.125	0.025	−0.016	0.790
Relative wall thickness	−0.096	0.089	−0.046	0.413	0.085	0.157
Hs-CRP	−0.120	0.065	0.035	0.589	0.118	0.093
Exercise duration	0.034	0.533	−0.003	0.953	−0.119	0.038
Metabolic equivalents	−0.004	0.943	−0.001	0.989	−0.144	0.012
Maximal SBP during exercise	−0.127	0.018	−0.065	0.222	−0.042	0.471
Maximal DBP during exercise	−0.008	0.887	−0.071	0.181	−0.081	0.161
24-hour mean SBP	−0.343	<0.001	0.139	<0.001	0.157	0.006
24-hour mean DBP	−0.255	<0.001	0.137	<0.001	0.005	0.927
24-hour mean heart rate	−0.149	0.005	0.129	0.015	0.025	0.666
24-hour mean BP SD	−0.251	<0.001	0.213	<0.001	0.148	0.010
Fasting glucose	−0.070	0.262	0.018	0.772	−0.040	0.548
Low density lipoprotein	0.044	0.463	−0.029	0.630	0.010	0.879
High density lipoprotein	−0.071	0.235	0.077	0.194	−0.081	0.208
Triglycerides	−0.036	0.543	−0.055	0.349	−0.042	0.514
eGFR MDRD	0.109	0.068	−0.033	0.579	−0.087	0.184
HRR	1		−0.720	<0.001	−0.309	<0.001
EFT	−0.309	<0.001	0.238	<0.001	1	

*Hs-CRP* high sensitivity C-reactive protein, *BP* blood pressure, *HR* heart rate, *SBP* systolic blood pressure, *DBP* diastolic blood pressure, *BP* blood pressure, *SD* standard deviation

**Table 6:** Binary regression analysis for the heart rate recovery in study groups

Predictors	Odd ratio	95 % confidence interval	*p*-value
Age	1.00	0.98 to 1.02	0.917
Body mass index	0.97	0.90 to 1.05	0.418
Epicardial fat thickness	3.53	1.20 to 10.37	0.022
Presence of diabetes mellitus	1.15	0.43 to 3.11	0.783
24-hour mean blood pressure variation	1.09	1.03 to 1.16	0.005
24-hour mean systolic blood pressure	1.00	0.97 to 1.04	0.859
24-hour mean diastolic blood pressure	0.99	0.95 to 1.03	0.644

## Discussion

This is the first study investigating the association between EFT and HRR from symptom-limited exercise testing in hypertensive patients according to the diurnal variation within our knowledge. The most relevant findings obtained from this study are as follows: 1) epicardial fat was thickest in the non-dipper group, 2) HRR was lower in hypertensive patients, 3) there was a significant correlation between EFT and HRR, and HRR was also significantly correlated with 24-hour mean systolic/diastolic BP variability and 4) EFT and circadian BP variability were independent predictors of blunted HRR in patients with hypertension. Our findings suggest a possible link between epicardial fat and autonomic dysregulation in hypertension.

Abnormalities of the autonomic nervous system play a fundamental role in BP regulation, and the majority of hypertensive patients have increased sympathetic activity along with diminished parasympathetic tone. Furthermore, the lack of a decrease in nocturnal BP is associated with severe end-organ damage and an increased risk of cardiovascular events, especially in hypertensive patients [1, 2]. Although the underlying mechanisms for a blunted nocturnal fall in BP are still uncertain, non-dippers are suggested to show impairment in the autonomic system that includes abnormal parasympathetic and increased sympathetic nervous system activity [3, 4, 23]. Therefore, a novel, non-invasive risk assessment tool that is affected by the same physiological mechanism as circadian BP rhythm would be useful to identify patients who might have impaired nocturnal BP patterns.

Recently, epicardial adipose tissue has been found to reflect visceral adiposity and has been proposed as a new cardiometabolic risk factor, carrying more risk than general fat accumulation [11–13]. Several reports have found a possible association between epicardial fat and hypertension, and increased EFT was independently associated with blunted nocturnal BP fall in hypertensive individuals [15, 16]. Increased plasma fatty acid levels may stimulate the cardiac autonomic nervous system through an increase in plasma catecholamine levels, which may be related to impaired diurnal BP patterns. Consequently, there might be a possible correlation between EFT and circadian BP variability in hypertensive patients.

Generally, an increase in HR during exercise occurs as a result of the combination of sympathetic activation and parasympathetic withdrawal. In contrast, parasympathetic reactivation is the principal determinant of the decrease in HR during early recovery, and this mechanism is independent of age and the intensity of exercise [24]. Given the prognostic significance of diminished parasympathetic tone at rest, post-exercise HRR is a noninvasive method that enables assessment of parasympathetic activation [24, 25]. Hence, we might estimate that parasympathetic “insufficiency” is implicated in the increased mortality risk in patients with abnormal HRR [8, 9, 24, 26]. Because HRR is simple to calculate from data obtained from standard exercise tests and does not require either 24-hour Holter monitoring or specialized baroreflex-sensitivity testing, HRR may be valuable for the assessment of risk in routine clinical practice. Considering non-dippers show impairment in the autonomic system that includes abnormal parasympathetic and increased sympathetic nervous system activity, we propose a correlation between blunted HRR and an increased EFT according to the diurnal variation. In our results, EFT was greatest in patients with a non-dipping BP pattern, and there was significant correlation between EFT and HRR, as expected. Although HRR was significantly correlated with 24-hour mean systolic/diastolic BP variability, HRR was slower in both hypertensive dipper and non-dipper groups compared to the normotensive controls. These findings suggest that an increase in cardiac sympathetic activity rather than diminished parasympathetic tone might be the dominant feature of the non-dipping pattern. Moreover, EFT and circadian BP variability were independent predictors of the blunted HRR in patients with hypertension, which implicates a link between epicardial fat and autonomic dysregulation in hypertension. In our study, we also found a significant association between EFT and exercise capacity. Considering the prognostic significance of blunted HRR or exercise capacity, our results might suggest a role of EFT in the adverse outcomes in hypertensive patients. However, prognostic implications of blunted HRR in this particular patient population was beyond the scope of our study.

Our study has several limitations. First, to decrease the effect of ischemic symptoms after the termination of exercise test, we excluded patients with a history of heart failure, revascularization, or a positive result of an exercise test. However, the presence of coronary artery disease was excluded using only exercise testing, and further stress imaging modalities were not used. Second, because this study was performed at a single tertiary care center, there might be biases in patient referral and population sampling. Moreover, previous hypertensive medications might have an important impact on the BP variability and HRR. In order to account for these possible confounding effects, we performed sensitivity analysis for the binary regression analysis according to the use or nonuse of medications; the results were not different. Finally, EFT can be affected by metabolic syndrome, however, we do not check the waist circumference from the enrolled patients, so the metabolic syndrome cannot be defined from this result. However, regarding the significant correlation between EFT and obesity represented by BMI or EFT and 24-hour mean SBP, we can imagine the possible association between metabolic syndrome and EFT.

## Conclusions

In conclusion, EFT, an indicator of cardiac autonomic activity, was greatest in hypertensive patients with a non-dipping pattern, and impaired HRR, an indicator of abnormal parasympathetic reactivation, was observed in hypertensive patients regardless of nocturnal BP patterns. There was a significant correlation between EFT and HRR, and EFT and circadian BP variability were independent predictors of blunted HRR in patients with hypertension. Our data suggest that there is a cross link between epicardial fat and autonomic dysregulation in hypertension. The association between EFT and adverse cardiovascular outcomes in patients with blunted HRR needs to be investigated in further detail in future research.
